# Subacute hemorrhagic pericardial tamponade after COVID-19 infection mimicking carcinomatous pericarditis: a case report

**DOI:** 10.3389/fcvm.2023.1329952

**Published:** 2024-01-09

**Authors:** Hiroyuki Yamamoto, Nao Kume, Katsuya Hashimoto, Jun Isogai, Takuya Kuwabara, Masayuki Noguchi, Hiroyuki Murayama, Toru Hashimoto, Hidemitsu Ogino

**Affiliations:** ^1^ Department of Cardiovascular Medicine, Narita-Tomisato Tokushukai Hospital, Chiba, Japan; ^2^ Department of Cardiology, Tokyo Medical University Hospital, Tokyo, Japan; ^3^ Department of Surgery, Narita-Tomisato Tokushukai Hospital, Chiba, Japan; ^4^ Division of Radiology, Asahi General Hospital, Asahi, Japan; ^5^ Department of Pathology, Narita-Tomisato Tokushukai Hospital, Chiba, Japan

**Keywords:** COVID-19, hemorrhagic pericardial tamponade, acute pericarditis, cytology, sarcoid-like reaction

## Abstract

**Background:**

Coronavirus disease (COVID-19)-associated acute pericarditis has recently received much attention owing to its high frequency associated with pericardial tamponade (PT), showing unfavorable prognosis. However, early diagnosis and treatment remain challenging in cases of non-specific signs and symptoms.

**Case presentation:**

A 64-year-old man was admitted to our hospital for acute osteomyelitis of the toes and was properly treated with antimicrobial agents. Three days after admission, the patient developed mild COVID-19 without pneumonia, for which early anti-COVID-19 agents were initiated. Nevertheless, the patient developed hemorrhagic PT due to acute pericarditis 2 weeks later, which was confirmed by cardiac magnetic resonance, requiring an urgent pericardiocentesis. Although cytological analysis of the hemorrhagic pericardial fluid strongly suggested adenocarcinoma, the atypical cells were eventually proven to be mesothelial cells with reactive atypia. Furthermore, lymph nodes swelling with abnormal 2-[18F]-fluoro-2-deoxy-D-glucose accumulation on imaging were suggestive of malignancy. However, biopsy examination revealed multiple non-caseating granulomas in the lymph node, unlikely due to malignancy. Eventually, the temporal association of the preceding COVID-19 with the occurrence of subacute PT without other identifiable cause led to a final diagnosis of COVID-19-associated acute pericarditis. With anti-inflammatory and corticosteroids treatment, the patient's symptoms involving the pericardial structure and function were completely resolved along with improvements in size of the affected lymphadenopathies.

**Conclusions:**

We encountered a unique case of COVID-19-associated acute pericarditis exhibiting hemorrhagic PT. This case underscores the residual risk of delayed pericardial involvement even in patients with mild COVID-19 who receive early treatment, and the recognition that COVID-19 may cause various cytomorphological and histological features. Additionally, the importance of considering this rare entity as a cause of hemorrhagic pericardial effusions should be highlighted.

## 1 Introduction

Coronavirus disease (COVID-19) due to severe acute respiratory syndrome coronavirus 2 (SARS-CoV-2) has led to a worldwide pandemic. Among extrapulmonary manifestations of COVID-19, acute pericarditis has recently gained attention owing to its high frequency associated with pericardial tamponade (PT), which is a life-threatening condition requiring prompt interventions (1–4). Acute pericarditis is diagnosed based on the following symptoms and signs: pleuritic chest pain, pericardial friction rub, typical changes in the electrocardiogram (ECG), and new-onset or worsening pericardial effusion (5). However, early diagnosis and treatment of acute pericarditis remain difficult in cases of non-specific signs and symptoms. Here, we report a unique case of subacute hemorrhagic PT after nosocomial COVID-19 infection, mimicking carcinomatous pericarditis.

## 2 Case description

**Figure 1:**
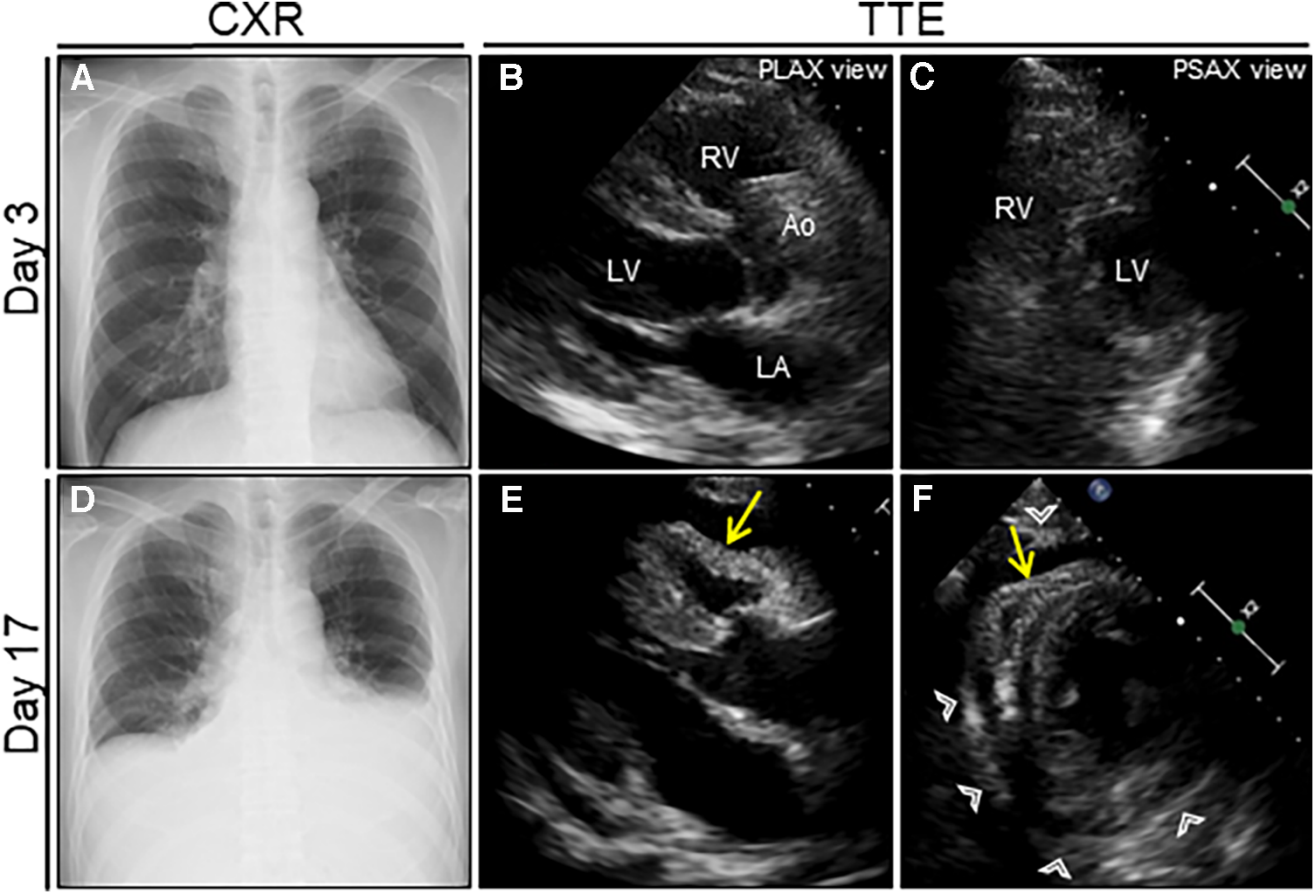
Serial chest radiograph (CXR) and transthoracic echocardiography (TTE) after nosocomial infection of COVID-19. On day 3 after admission (1 day after nosocomial infection of SARS-CoV-2), CXR (**A**) and TTE (**B, C**) are unremarkable. On day 17 (15 days after nosocomial infection of SARS-CoV-2), the follow-up CXR reveals cardiac enlargement with bilateral pleural effusions (**D**), whereas the follow-up TTE reveals a moderate pericardial effusion with pericardial thickening (arrowheads) (**E, F**). Note the right ventricular collapse during early diastole (arrows). Ao, aorta; COVID-19, coronavirus disease; LA, left atrium; LV, left ventricle; PLAX, parasternal long-axis; PSAX, parasternal short-axis; RV, right ventricle; SARS-CoV-2, severe acute respiratory syndrome coronavirus 2.

**Figure 2:**
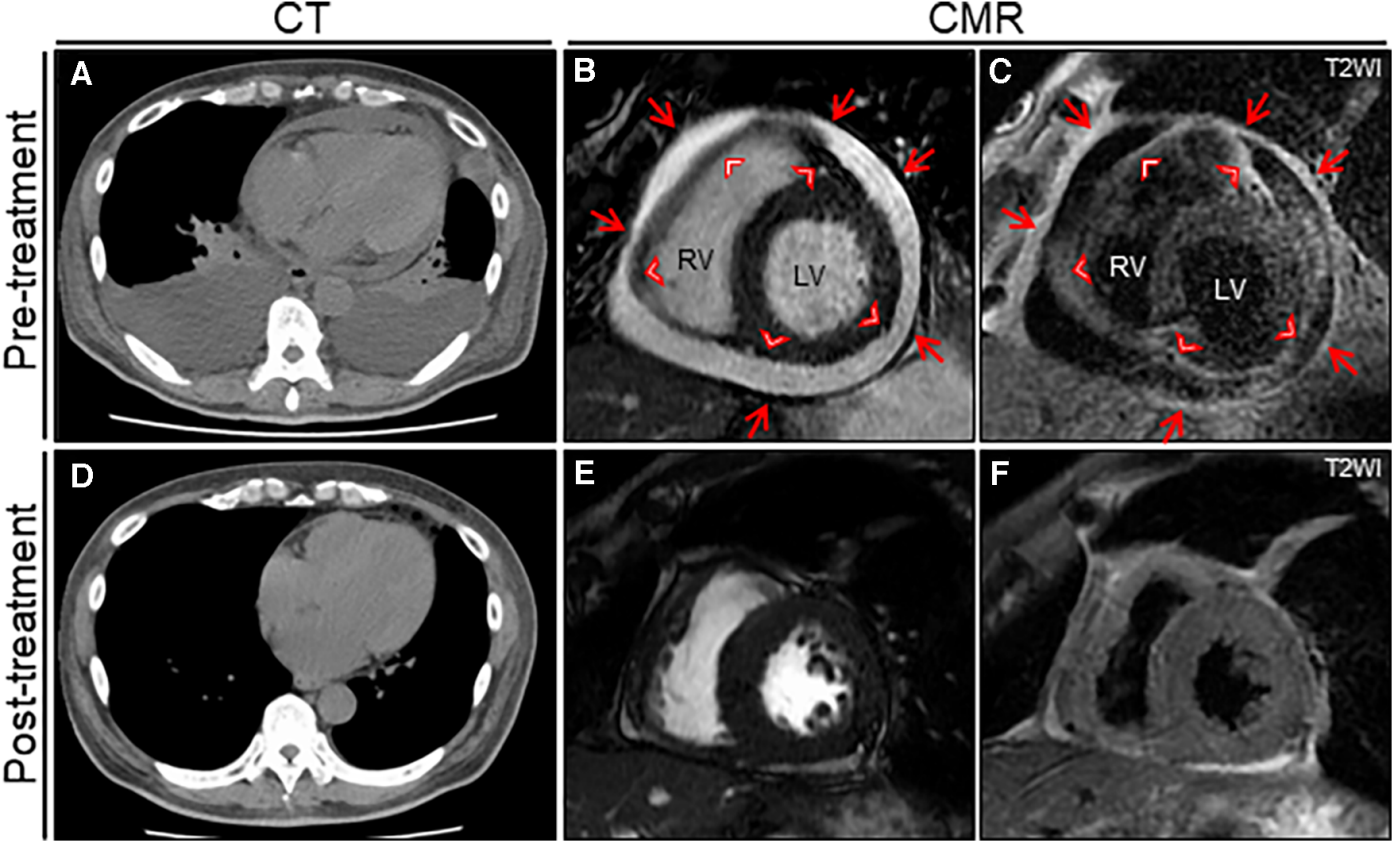
The treatment effect on chest computed tomography (CT) and cardiac magnetic resonance (CMR) findings. Chest CT reveals moderate pericardial and bilateral pleural effusions with passive atelectasis (**A**) that resolves completely at the 6-month follow-up (**D**) Cine CMR reveals a moderate pericardial effusion at baseline (**B**) that resolves significantly at the 6-month follow-up (**E**) Note the entire thickening of epicardium (arrowheads) and pericardium (arrows). T2-weighted image (T2WI) shows active diffuse pericardial edema of the epicardium (arrowheads) and pericardium (arrows) observed at baseline (**C**) that resolves significantly at the 6-month follow-up (**F**). LV, left ventricle; RV, right ventricle.

**Figure 3:**
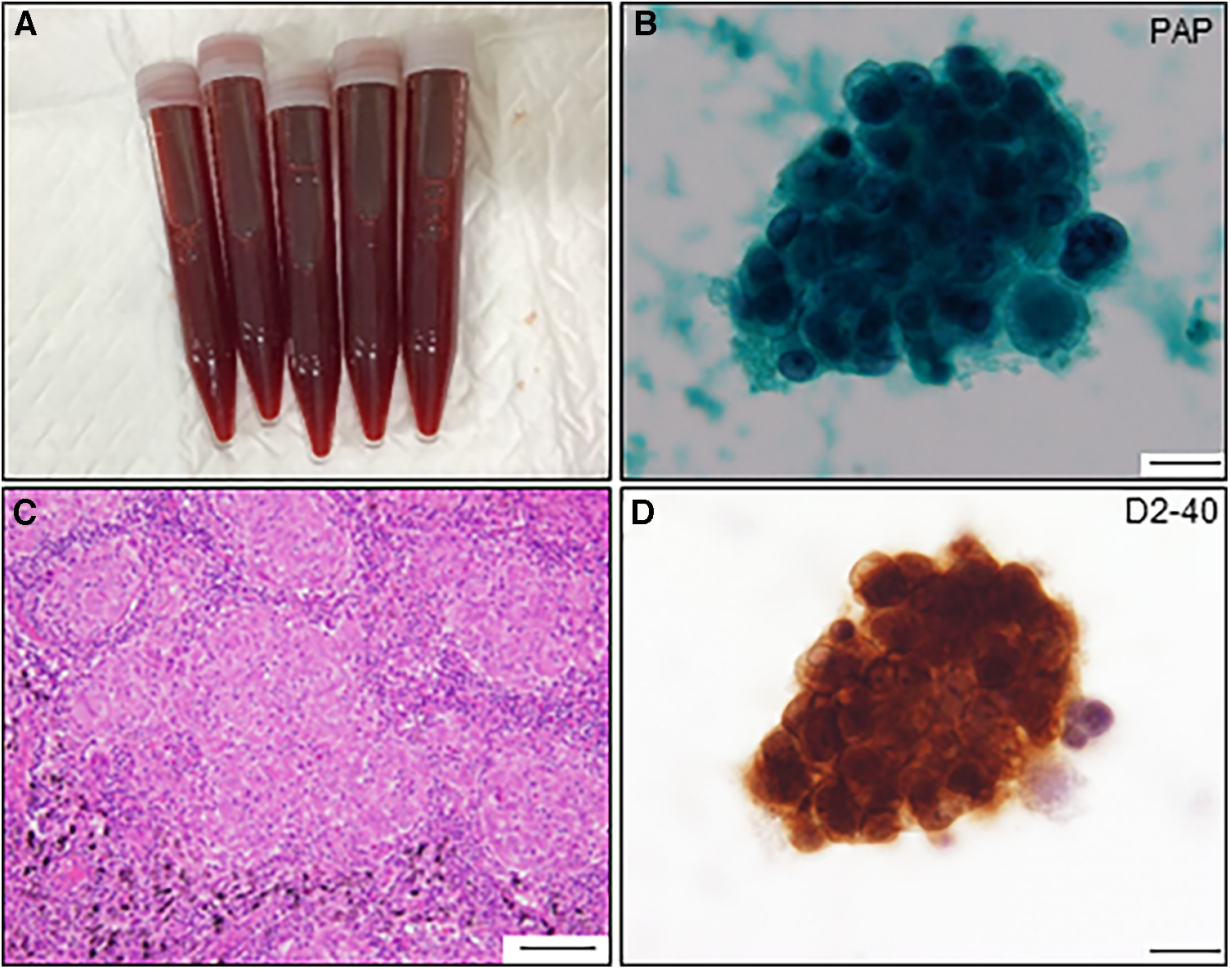
Pericardial fluid and cytological findings and histological feature of resected lymph node. (**A**) Pericardial fluid shows hematogenous appearance. (**B**) Atypical cell nest is detected in the pericardial fluid and initially diagnosed as adenocarcinoma by Papanicolaou (PAP) staining (scale bar, 20 μm). (**C**) The resected lymph node shows multiple non-caseating granulomas (scale bar, 100 μm). (**D**) The same atypical cells observed in (**B**) are positive for anti-D2-40 antibody by immunocytochemistry (scale bar, 20 μm).

**Figure 4:**
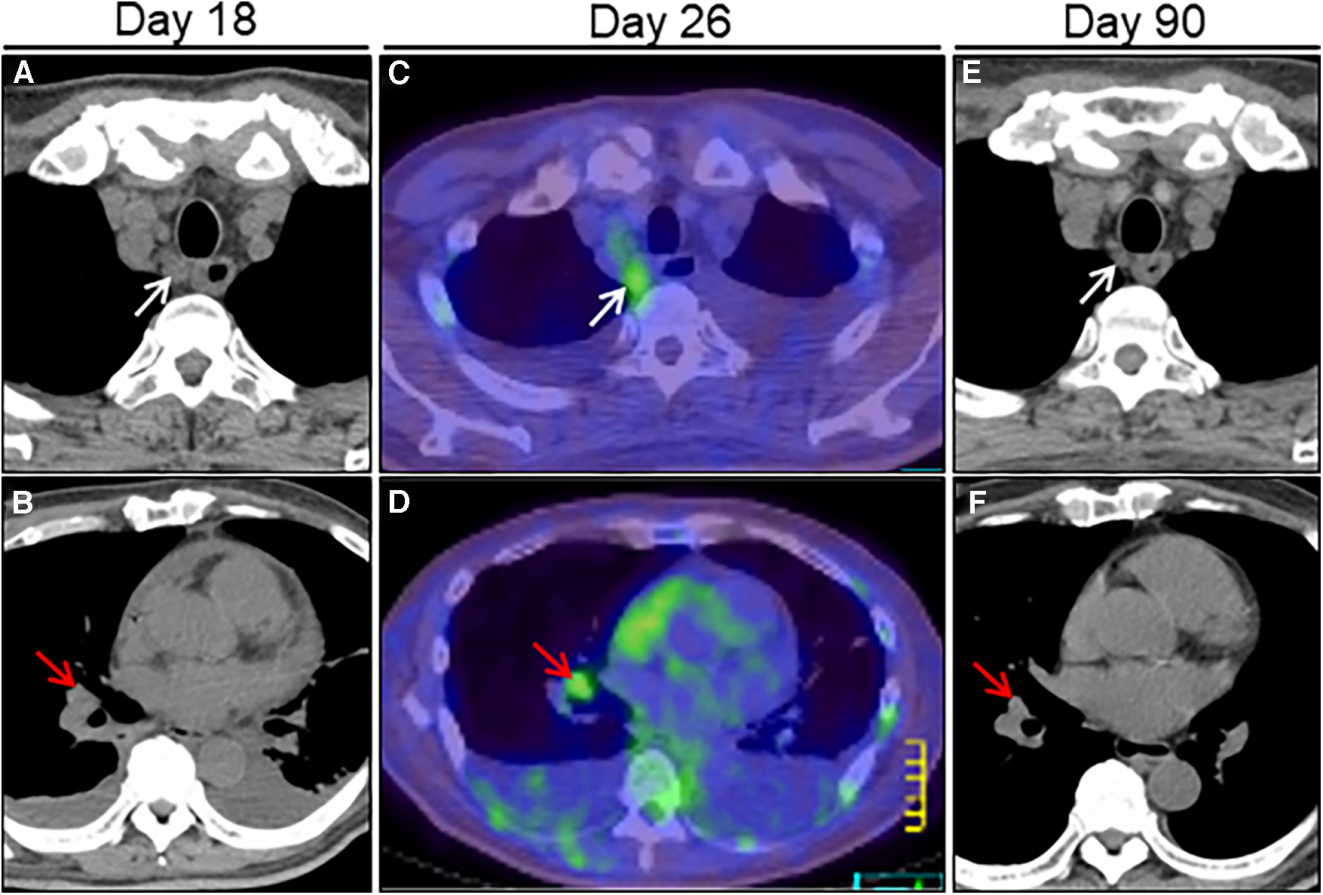
Treatment effect on lymphadenopathy on chest computed tomography (CT) imaging. Chest CT on day 18 after admission reveals right paraesophageal (white arrow) and hilar (red arrow) slight lymphadenopathies (**A, B**). FDG/PET-CT on day 26 reveals slight hypermetabolic activities in the same ones (**C, D**). Post-treatment follow-up CT on day 90 reveals a significant improvement in size of the affected lymphadenopathies (**E, F**). FDG/PET, positron emission tomography with 2-[18F]-fluoro-2-deoxy-D-glucose.

**Figure 5:**
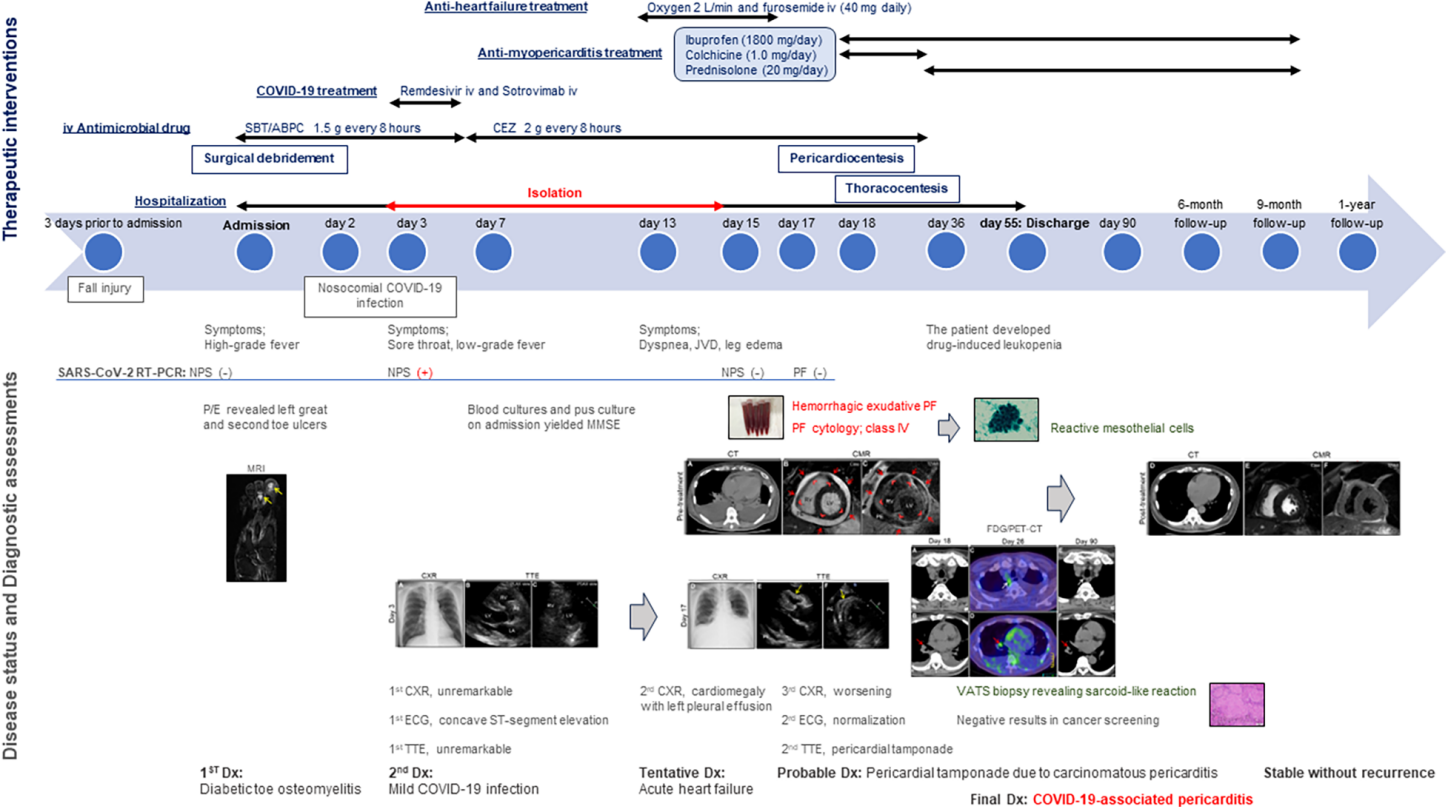
Timeline of the diagnostics, therapeutic interventions, and disease status of the present case. CEZ, cephazolin; CMR, cardiac magnetic resonance; CT, computed tomography; CXR, chest radiograph; COVID-19, coronavirus disease; Dx, diagnosis; ECG, electrocardiogram; FDG/PET, glucose analog 2-[18F]-fluoro-2-deoxy-d-glucose positron emission tomography; iv, intravenous; JVD, jugular vein distention; MMSE, methicillin-sensitive *Staphylococcus aureus*; MRI, magnetic resonance imaging; NPS, nasopharyngeal swab; P/E, physical examination; PF, pericardial fluid; RT-PCR, reverse-transcriptase polymerase chain reaction; SARS-CoV-2, severe acute respiratory syndrome coronavirus 2; SBT/ABPC, ampicillin-sulbactam; TTE, transthoracic echocardiography; VATS-biopsy, video-assisted thoracoscopic biopsy.

A 64-year-old man was admitted to our hospital for acute osteomyelitis of the toes, presenting with left toes ulcer due to infection developed after a fall injury 3 days prior to admission. The patient had a past medical history of hypertension, chronic kidney disease (CKD), and diabetes mellitus controlled using insulin. The patient's initial vital signs were as follows: blood pressure, 189/104 mmHg; heart rate, 109 beats/min; and blood temperature, 39.8°C. Physical examination of his left great and second toes revealed ulcers with pus, swelling, and surrounding erythema. Laboratory test results revealed white blood cell count, 10,000 cells/µl (differential count, 88.9% neutrophils); elevated C-reactive protein levels, 3.09 mg/dl (normal: <0.14 mg/dl); fasting blood glucose, 316 mg/dl (normal: <110 mg/dl); and glycated hemoglobin, 12.4% (normal: <6.0%). Moderate renal dysfunction was observed. SARS-CoV-2 was undetectable using reverse-transcriptase polymerase chain reaction (RT-PCR) on a nasopharyngeal swab specimen. Magnetic resonance imaging (MRI) indicated acute osteomyelitis of the toes (Supplementary Figure S1). Subsequently, the patient underwent surgical debridement of the ulcers to enhance the healing process. Following the collection of blood and pus for cultures, the patient was intravenously administered with ampicillin-sulbactam (1.5 g every 8 h). Simultaneously, a continuous insulin infusion was initiated to strictly control hyperglycemia. On the second day after admission, nosocomial transmission of SARS-CoV-2 infection occurred. A repeated nasopharyngeal swab for RT-PCR showed negative results; however, on day 3, the patient complained of a sore throat. His vital signs were stable except for low-grade fever of 36.9°C. A follow-up RT-PCR showed positive results for SARS-CoV-2. His physical examination and chest radiograph showed unremarkable findings (Figure 1A). Serum cardiac enzyme levels were within normal ranges. ECG showed sinus rhythm and concave ST-segment elevation in precordial leads, suggestive of early repolarization (Supplementary Figure S2A). Echocardiography showed mild concentric left ventricular (LV) hypertrophy with normal contraction and enlarged left atrium suggestive of diastolic dysfunction; however, pericardial effusion was not observed (Figures 1B,C and Supplementary Videos S1, S2). After the patient was isolated, intravenous infusions of antiviral agent (remdesivir, a loading dose of 200 mg followed by 100 mg for 2 days) and SARS-CoV-2 neutralizing antibody (sotrovimab, a single dose of 500 mg) were administered for mild COVID-19 treatment. On day 7, all the cultures collected on admission yielded *Staphylococcus aureus* sensitive to cefazolin and de-escalation of intravenous cephazolin (2 g every 8 h) was performed for 4 weeks. The patient achieved improvement in glycemic control and was then switched to conventional regular subcutaneous insulin. His wound healing process was uneventful. On day 13, the patient experienced dyspnea. His vital signs were blood pressure, 134/77 mmHg; heart rate, 68 beats/min; body temperature, 36.7°C; respiratory rate, 18 breaths/min; and oxygen saturation, 85% on ambient air. A follow-up chest radiograph revealed cardiomegaly with left pleural effusion. The patient's body weight increased by 8 kg. Jugular vein distention and bilateral leg edema were noted; serum brain natriuretic peptide level was elevated (332 pg/ml, normal: <18 pg/ml). Therefore, a presumptive diagnosis of acute heart failure was made, and the patient was administered oxygen at 2 L/min and treated with intravenous loop diuretics (furosemide, 20 mg twice daily) to control volume overload. On day 15, a repeated RT-PCR confirmed SARS-CoV-2 negativity, ending isolation. On day 17, the patient's condition exacerbated (Figure 1D). Chest computed tomography (CT) revealed moderate pericardial and bilateral pleural effusions (Figure 2A). The follow-up ECG showed ST-segment normalization (Supplementary Figure S2B). The follow-up echocardiography revealed a moderate pericardial effusion with tamponade physiology, suggestive of PT (Figures 1E,F and Supplementary Videos S3, S4). Cardiac MRI suggested active pericarditis (Figures 2B,C). Right heart catheterization confirmed PT with equalization of diastolic pressures across all chambers and marked hemodynamic pulsus paradoxus (Supplementary Table S1). Subsequently, the patient underwent an urgent pericardiocentesis with a placement of pericardial drainage, showing 750 ml of hemorrhagic exudate fluid (Figure 3A). Pericardial fluid (PF) analysis showed hypercytokinemia consistent with an inflammatory process (Supplementary Table S2). SARS-CoV-2 in the PF was undetectable using RT-PCR. Gram and Ziehl-Neelsen staining and bacterial and fungal cultures yielded negative results. Serologic testing for autoimmune diseases and cardiotropic viruses workup indicated negative results. Notably, PF cytology suggested adenocarcinoma cells suspecting carcinomatous pericarditis (Figure 3B), for which ibuprofen (600 mg three times daily) and colchicine (0.5 mg twice daily) were initiated. On day 18, the patient underwent bilateral thoracocentesis with drainage of serous transudate pleural fluids, which also showed hypercytokinemia (Supplementary Table S3). SARS-CoV-2 in the pleural fluids was also undetectable using RT-PCR. After confirmation of no pericardial effusion recurrence, the drain was removed. Upper endoscopy and colonoscopy for cancer screening showed unremarkable findings. CT screening revealed right paraesophageal and hilar lymph nodes swelling (Figures 4A,B). On day 26, positron emission tomography (PET)/CT with the glucose analog 2-[18F]-fluoro-2-deoxy-d-glucose (FDG) disclosed the slight hypermetabolic activities in the same lesions (Figures 4C,D). Owing to the concern for malignancy, the patient underwent video-assisted thoracoscopic biopsy (VATS-biopsy) of the FDG-avid hilar lymph node, revealing multiple non-caseating granulomas suggestive of sarcoidosis (Figure 3C). However, considering normal serum angiotensin converting enzyme (ACE) levels and the absence of pulmonary, skin, and eye involvement, the patient was diagnosed with a sarcoid-like reaction. Furthermore, re-examination of the PF cytological materials using immunohistochemistry revealed that the atypical cells were reactive mesothelial cells because they were positive for D2-40, a specific cell-marker for mesothelial cell (Figure 3D), which was unlikely due to malignancy. Therefore, based on the temporal association between preceding SARS-CoV-2 infection and the development of subacute PT without other identifiable causes, a final diagnosis of COVID-19-associated acute pericarditis was made. On day 36, the patient developed leukopenia during treatment, which was suspected to be drug-induced, that resolved with colchicine discontinuation. Alternatively, oral prednisolone (20 mg/day) was added because of residual pericardial thickening with pericardial effusion on the follow-up echocardiography. Thereafter, the patient's clinical condition improved steadily, and he was discharged on day 55. A significant improvement in size of the affected lymphadenopathies was also observed on day 90 (Figures 4E,F). Furthermore, a complete resolution of pericardial structural and functional abnormalities with concurrent pleural effusions was observed at the 6-month follow-up (Figures 2D–F). Thereafter, ibuprofen and prednisolone were tapered and discontinued over 3 months. The patient remains clinically stable during the first year of follow-up. We present a summarized illustration of the case presentation in Figure 5.

## 3 Discussion

COVID-19 was first reported in Wuhan, China, in 2019 and became a global pandemic. Although COVID-19 primarily affects the respiratory system, which commonly progresses to acute respiratory distress syndrome in severe cases, it can affect all organs (6). Regarding the cardiovascular system, pericardial involvement has increasingly gained attention with growing pieces of evidence (3). The accurate incidence of pericardial involvement remains unknown. However, a retrospective multicenter study suggested the potential high risk of acute pericarditis in patients with COVID-19 compared with those without COVID-19 (odds ratio, 1.45; 95% confidence interval, 1.07–1.97) (7). Furthermore, COVID-19-associated acute pericarditis has been presumed to exhibit a more aggressive phenotype compared with acute pericarditis in the pre-COVID-19 pandemic era owing to the higher frequency of associated pleural effusions (76%) and PT (35%), with poor prognosis (1, 8). Herein, we report a case of subacute hemorrhagic PT after nosocomial COVID-19 infection, successfully treated with pericardiocentesis followed by medical treatment. Our case may provide four valuable clinical lessons.

First, this is the first report of COVID-19-associated pericarditis mimicking malignancy. Our patient had to be differentiated from carcinomatous pericarditis based on the following three findings.

As the first finding, our case presented with hemorrhagic PT. Although causative diseases underlying hemorrhagic PT varies with the era, region, and patient population, a retrospective observational study showed that most hemorrhagic PT causes included invasive cardiac surgery-related (31%), malignancy (26%), mechanical complications of myocardial infarction (10%), and idiopathic etiologies (10%). Miscellaneous causes include tuberculosis, trauma, uremia, aortic dissection, drugs (e.g., anticoagulant or anti-TNF alpha agent), and viral infection (9–11). Generally, viruses have been considered rare etiologies for hemorrhagic pericardial effusions, with the exception of Coxsackievirus (12). However, given the certain incidence of previous case reports and case series with COVID-19 presenting with hemorrhagic pericardial effusion (4, 13–15), our case highlighted the significance of considering COVID-19 as an etiology of hemorrhagic pericardial effusion.

The second finding was the PF cytology result suggesting adenocarcinoma. However, re-examination of immunostaining with D2-40 revealed mesothelial cells with reactive atypia. These phenomena are most likely due to the aberrant hyperimmune response to cytokine storm during acute infection. A previous study evaluated the impact of COVID-19 on cytomorphological manifestations of mesothelial cells in body fluids. It revealed that more atypical mesothelial cells having multinucleation, bizarre nuclei, and prominent nucleoli (73.9% vs. 53.8%, *p* < 0.005), requiring more immunostains ordered (47.8% vs. 7.7%, *p* < 0.014), were found in body fluids during the active phase compared with those during the recovery phase (16), supporting this notion. Hence, this case emphasized the careful comprehension of cytomorphological changes in effusion fluid cytology associated with COVID-19.

The last finding was the presence of swollen lymph nodes on imaging. Metastatic lymph nodes associated with carcinomatous pericarditis were initially suspected based on the above two findings. However, VATS-biopsy of the FDG-avid hilar lymph nodes indicated non-caseating granulomas. Similar to the present case, there is a growing number of case reports of *de novo* sarcoidosis or sarcoid-like reactions in multiple organs induced by COVID-19 (17–19). Sarcoidosis and sarcoid-like reactions are histologically indistinguishable. Sarcoidosis is a multisystem disorder characterized by the accumulation of non-caseating granulomas with lymphocytic inflammation involving various organs including the lungs, skin, eyes, heart, and lymph nodes. Although the exact etiology and mechanism of sarcoidosis remain poorly understood, plausible mechanisms include hyperinflammatory immune-mediated responses to an antigen against infectious or environmental exposures. Previous studies have proposed a possible signal crosstalk between COVID-19 and sarcoidosis, which shared cell signaling pathways such as renin-angiotensin signaling, inhibited autophagy, and induced cell apoptosis (20). Similarly, COVID-19 might have induced sarcoid-like reaction in the lymph nodes in our case. Clinicians should recognize possible lymph node involvement after infection with COVID-19.

In summary, our case underscored characteristic cytomorphological and histological features caused by COVID-19.

As the second clinical lesson, early treatment of mild COVID-19 did not inhibit ongoing active pericarditis.

Approximately 80% of patients with COVID-19 have mild to moderate severity with or without pneumonia, and 15% of them progress to a severe or critical state without treatment (21). Given that viral load is a determinant of subsequent severity and prognosis, early pharmacological treatment to reduce viral load has a beneficial effect not only in reducing disease severity but also in controlling infection spread to multiple organs. Our patient had a history of hypertension, diabetes mellitus, and CKD, which are well-known risk factors for COVID-19 severity; thus, drug therapy was initiated immediately after the onset of mild COVID-19. Nevertheless, the patient developed ongoing pericarditis, resulting in hemorrhagic PT. Although the exact mechanism underlying pericardial involvement remains poorly understood, several mechanisms have been proposed (3, 4, 22). First, SARS-CoV-2 can directly infect cardiovascular tissue, such as cardiomyocytes, pericytes, endothelial cells, and macrophages, which express the ACE2 receptor. This infection can activate the ACE2 signaling pathway and result in cardiovascular injury. SARS-CoV-2 has actually been detected in the PF of a COVID-19 patient with pericarditis using RT-PCR (23), although the direct causal relationship remains unknown. Second, the normal pericardium, a double-layered membrane surrounding the heart composed of an inner visceral and outer parietal layer, is relatively avascular. However, pericarditis is characterized by hypervascularity. Under situations of vascular injury and inflammation triggered by SARS-CoV-2 infection, the ensuing vascular endothelial dysfunction in the pericardium may lead to pericarditis. Third, an aberrant hyperimmune response to cytokine storm following SARS-CoV-2 infection may lead to apoptosis of epithelial or endothelial cells and vascular damage with subsequent pericarditis. Fourth, SARS-CoV-2 infection primarily affects the lungs and often complicates acute respiratory distress syndrome. Therefore, oxidative stress caused by severe hypoxia can contribute to concomitant pericardial injury, leading to pericarditis. Given hypercytokinemia in the PF despite early COVID-19 treatment, the indirect systemic hyperinflammatory response to cytokine storm may be the crucial mechanism underlying pericardial involvement in our case. Hence, this case highlighted the importance of considering the residual risk of subsequent pericardial involvement even in patients with mild COVID-19 undergoing early pharmacological treatment.

For the third clinical lesson, T2-weighted cardiac MRI was also effective in diagnosing atypical acute pericarditis as per our case.

Acute pericarditis is diagnosed based on the presence of more than two of the following: (i) typical pleural chest pain; (ii) pericardial friction rub; (iii) generalized ST-segment elevation with reciprocal ST-segment depression and PR-segment elevation in leads aVR and V1 on ECG; and (iv) new-onset or worsening pericardial effusion (24). However, our case did not meet the above criteria except for new-onset pericardial effusion. Although contrast-enhanced CT/MRI was not available owing to renal dysfunction in our case, T2-weighted cardiac MRI could characterize pericardial inflammation, leading to the correct diagnosis of acute pericarditis. However, the early repolarization pattern on ECG observed on day 3 after admission might have been the initial manifestation of acute pericarditis in our case. Cardiac MRI can provide detailed morphological and functional evaluation of the pericardium (24). Therefore, cardiac MRI may be the first screening tool in patients suspected with acute pericarditis that is inconclusive based on the above-mentioned criteria.

The fourth clinical lesson is that ibuprofen and corticosteroids were effective in treating COVID-19-associated hemorrhagic pericarditis in our case.

Untreated hemorrhagic pericarditis may pose transition risk to chronic pericarditis or constrictive pericarditis. However, there is no current guideline for managing COVID-19-associated pericarditis. Although nonsteroidal anti-inflammatory drugs (NSAIDs) including ibuprofen and colchicine are the first line agents for acute viral pericarditis (5), there are the following two theoretical concerns of using NSAIDs for COVID-19-associated pericarditis. As a first concern, NSAIDs may involve in the upregulation of ACE2 protein, a known cell entry receptor for SARS-CoV-2, leading to potential risks of SARS-CoV-2 re-infection or COVID-19 severity. However, there is conflicting experimental data: ibuprofen augmented ACE2 expression in a rat model with diabetes (25), whereas ibuprofen had no impact on ACE2 expression and SARS-CoV-2 viral entry in human cell cultures (26). In addition, a large observational study revealed no significant relationship between NSAIDs use and COVID-19 occurrence or severity (27). As a second concern, NSAIDs have a potential risk of promoting hemorrhagic pericardial effusion owing to its antiplatelet activity. However, such association has not been observed in previous case reports (4, 28). Based on these findings, there is currently no solid evidence to prove that NSAIDs use could have detrimental effects on COVID-19 occurrence or severity. Corticosteroids are a potential therapeutic option; however, routine administration of corticosteroids for acute viral pericarditis treatment is fundamentally not recommended because corticosteroids may delay viral clearance in host cell, resulting in ongoing inflammation (5). Nevertheless, corticosteroids may be beneficial for certain cases with recurrent or resistant to the first-line agents, in which hyperinflammatory response could be detrimental to the host. The present case had to avoid receiving the continuous use of colchicine owing to drug-induced leukocytopenia and required corticosteroids as an alternative, which was eventually effective. The guidelines for management of COVID-19-associated acute pericarditis would be established in the future.

Finally, our case presented a hemorrhagic phenotype in a pericardial lesion. Similar phenomena have been reported in several case reports and case series involving various organs and tissues including the brain, retina, pancreas, gastrointestinal tract, arteries, and muscle (29–34). These facts strongly suggest unresolved common mechanisms underlying the hemorrhagic phenotype after infection with COVID-19. Blood vessels are primarily composed of two interacting cell types: endothelial cells and perivascular cells (pericytes, vascular smooth muscle, or mural cells). Endothelial cells form the inner layers of the vessel wall, whereas perivascular cells encase the surface of the vessel wall. Importantly, endothelial cell/pericyte interactions are critical to maintain the homeostasis of the microvasculature, including vessel remodeling or angiogenesis (35).

Given the high ACE2 expression in human cardiac pericytes, these cells may be an attractive target for SARS-CoV-2. A study demonstrated that SARS-CoV-2 directly infects cardiac pericytes in patients with COVID-19-associated myocarditis, leading to the upregulation of inflammatory chemokines, cytokines, type I interferon, and vasoactive mediator genes with subsequent endothelial inflammation, in addition to enhancing the death of infected pericytes, which is dependent on the nuclear factor-kappa B pathway (36). During SARS-CoV-2 infection, microvascular thrombosis and cytokine storm can occur frequently, causing inflammation and endothelial cell injury, as well as vascular permeability. Therefore, the failure of functional and anatomical interactions between endothelial cells and pericytes may challenge vessel integrity, resulting in serious hemorrhagic phenotype after COVID-19 infection. A case report of ongoing hemorrhagic pericarditis, requiring the complete removal of the epicardium, revealed neovascularization and inflammation on histology, suggesting spontaneous rupture of microvasculature as a possible etiology (37). Similarly, a CMR imaging study of histological analyses of excised pericardium from patients with active constrictive pericarditis emphasized the importance of chronic inflammation and neovascularization during the active phase of pericarditis (38). These findings suggest that the failure of neovascularization, caused by dysregulated endothelial cell/pericyte interactions, might be a possible etiology of hemorrhagic pericarditis associated with COVID-19 infection.

Reports have indicated that the ongoing evolution of SARS-CoV-2 variants is associated with various cardiovascular diseases (39). The acute phase of COVID-19 may involve cardiac manifestations including acute coronary syndrome, myocarditis, pericarditis, heart failure, and arrhythmias. In addition, vascular involvements include acute venous (pulmonary embolism and deep vein thrombosis) and arterial (stroke and critical limb ischemia) thromboses, bleeding, coagulopathy, or disseminated intravascular coagulation. Notably, post-acute sequelae of COVID-19 infection cause long-term adverse cardiovascular events including stroke, heart failure, and arrhythmias. Although SARS-CoV-2 variants of concern have mutations that increase transmissibility and potentially worsen disease severity, their impact on cardiovascular involvement and patient outcomes remains poorly understood. Further evidence is warranted.

The present case report had three limitations. First, the definitive diagnosis of viral pericarditis was originally established based on a histological evaluation of the pericardium and viral genomic analyses with pericardial tissue specimen using RT-PCR. In the present case, pericardiectomy was not required since pericardial involvement showed complete recovery with medical treatment. Therefore, we could not reveal the direct causal relationship between COVID-19 and the development of acute pericarditis. In addition, the hypothesized mechanism of the hemorrhagic phenotype after COVID-19 infection could not be confirmed due to the lack of histology of the resected pericardium. Second, there was no solid evidence of causal link between COVID-19 and sarcoid-like reaction owing to the lack of the imaging information on FDG/PET-CT prior to COVID-19 and detailed immunohistological examination of the lymph node in our case. Third, a COVID-19 variant was not determined by genotyping in our case. Therefore, it remains unclear whether the delayed hemorrhagic pericarditis in this case is a complication specific to SARS-CoV-2 variants of concern.

## 4 Conclusions

Herein, we describe a case of COVID-19-associated acute pericarditis complicated by hemorrhagic PT. PT can be a fatal sequela of untreated acute pericarditis. However, COVID-19-associated acute pericarditis may sometimes mimic malignancy, leading to delayed diagnosis and treatment. Therefore, clinicians should recognize the pericardium as an important target of cardiovascular involvement and cytomorphological and histological features after COVID-19 infection. Accordingly, clinicians should consider this rare clinical entity in the diagnostic workup of hemorrhagic pericardial effusion.

## Data Availability

The original contributions presented in the study are included in the article/Supplementary Material, further inquiries can be directed to the corresponding author.

## Supplementary material

Supplementary Figure S1 Magnetic resonance imaging (MRI) of the left foot. T2-weighted short tau inversion recovery MRI reveals high signal intensity spots in the left distal phalanx of first toe and the proximal phalanx of second toe (arrows).

Supplementary Figure S2 Serial electrocardiogram (ECG) on day 3 (**A**) and on day 17 (**B**) after admission (**A**) ECG shows concave ST-segment elevation in precordial leads (V1-4). Note the absence of reciprocal ST-segment depression and PR-segment elevation in leads aVR and V1. (**B**) ECG shows ST-segment normalization observed in (**A**) and T-wave inversions in leads II, III, aVF, V5, V6.

Supplementary Table S1 Right heart catheter measurements before and after pericardiocentesis.

Supplementary Table S2 Blood and pericardial fluid analyses.

Supplementary Table S3 Blood and pleural fluid analyses.

Supplementary Video S1 Transthoracic echocardiography on day 3 after admission: parasternal long-axis view.

Supplementary Video S2 Transthoracic echocardiography on day 3 after admission: parasternal short-axis view.

Supplementary Video S3 The follow-up transthoracic echocardiography on day 17: parasternal long-axis view.

Supplementary Video S4 The follow-up transthoracic echocardiography on day 17: parasternal short-axis view.
