# Supplementary figures and images for: Subacute hemorrhagic pericardial tamponade after COVID-19 infection mimicking carcinomatous pericarditis: a case report
Source: Front Cardiovasc Med. 2024 Jan 9;10:1329952. doi: 10.3389/fcvm.2023.1329952 (PMC10803410; doi:10.3389/fcvm.2023.1329952)

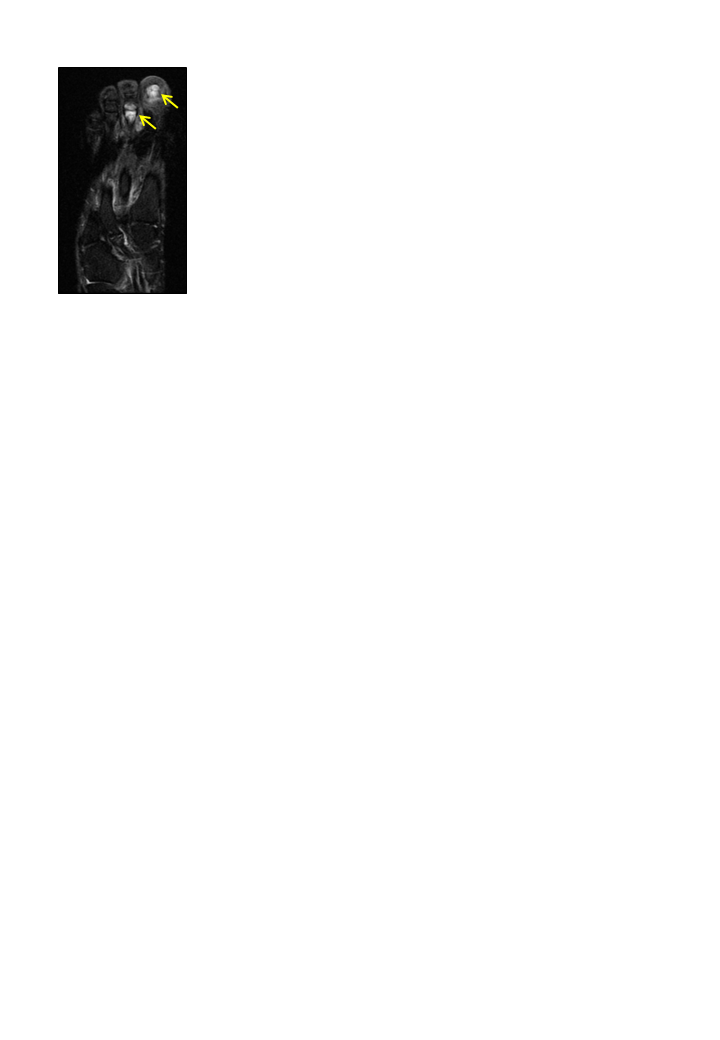

Supplement: Supplementary Figure S1 — Magnetic resonance imaging (MRI) of the left foot. T2-weighted short tau inversion recovery MRI reveals high signal intensity spots in the left distal phalanx of first toe and the proximal phalanx of second toe (arrows). [file Image1.tif]

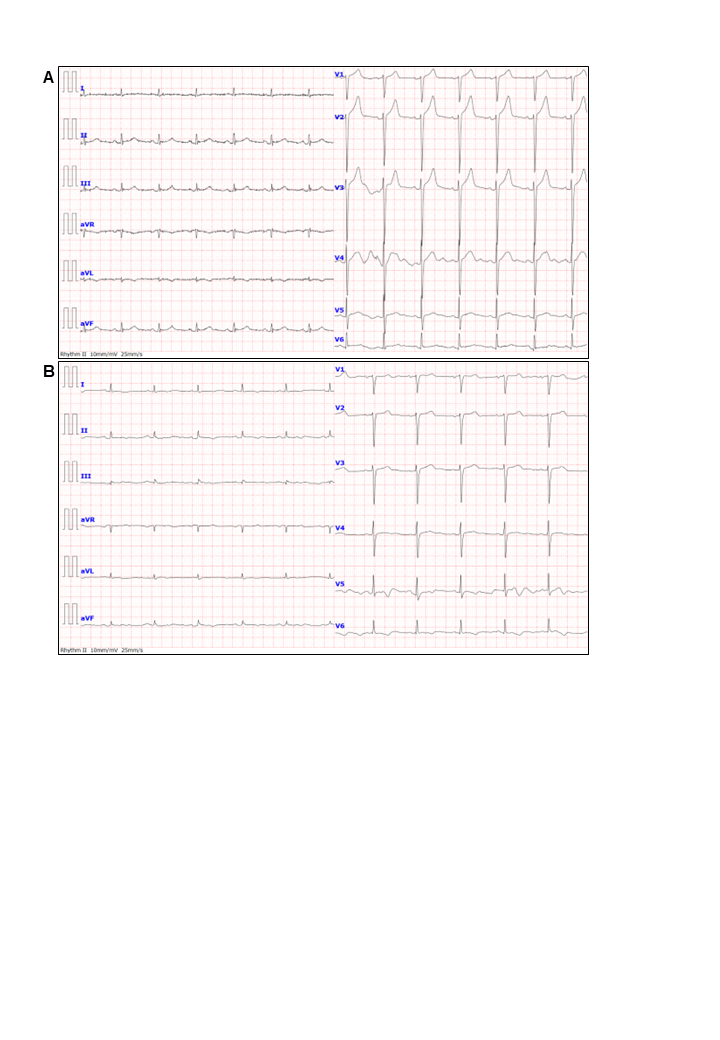

Supplement: Supplementary Figure S2 — Serial electrocardiogram (ECG) on day 3 (A) and on day 17 (B) after admission (A) ECG shows concave ST-segment elevation in precordial leads (V1-4). Note the absence of reciprocal ST-segment depression and PR-segment elevation in leads aVR and V1. (B) ECG shows ST-segment normalization observed in (A) and T-wave inversions in leads II, III, aVF, V5, V6. [file Image2.tif]
